# A novel scoring function for discriminating hyperthermophilic and mesophilic proteins with application to predicting relative thermostability of protein mutants

**DOI:** 10.1186/1471-2105-11-62

**Published:** 2010-01-28

**Authors:** Yunqi Li, C Russell Middaugh, Jianwen Fang

**Affiliations:** 1 Applied Bioinformatics Laboratory, the University of Kansas, Lawrence, KS 66047, USA; 2 Department of Pharmaceutical Chemistry, the University of Kansas, Lawrence, KS 66047, USA

## Abstract

**Background:**

The ability to design thermostable proteins is theoretically important and practically useful. Robust and accurate algorithms, however, remain elusive. One critical problem is the lack of reliable methods to estimate the relative thermostability of possible mutants.

**Results:**

We report a novel scoring function for discriminating hyperthermophilic and mesophilic proteins with application to predicting the relative thermostability of protein mutants. The scoring function was developed based on an elaborate analysis of a set of features calculated or predicted from 540 pairs of hyperthermophilic and mesophilic protein ortholog sequences. It was constructed by a linear combination of ten important features identified by a feature ranking procedure based on the random forest classification algorithm. The weights of these features in the scoring function were fitted by a hill-climbing algorithm. This scoring function has shown an excellent ability to discriminate hyperthermophilic from mesophilic sequences. The prediction accuracies reached 98.9% and 97.3% in discriminating orthologous pairs in training and the holdout testing datasets, respectively. Moreover, the scoring function can distinguish non-homologous sequences with an accuracy of 88.4%. Additional blind tests using two datasets of experimentally investigated mutations demonstrated that the scoring function can be used to predict the relative thermostability of proteins and their mutants at very high accuracies (92.9% and 94.4%). We also developed an amino acid substitution preference matrix between mesophilic and hyperthermophilic proteins, which may be useful in designing more thermostable proteins.

**Conclusions:**

We have presented a novel scoring function which can distinguish not only HP/MP ortholog pairs, but also non-homologous pairs at high accuracies. Most importantly, it can be used to accurately predict the relative stability of proteins and their mutants, as demonstrated in two blind tests. In addition, the residue substitution preference matrix assembled in this study may reflect the thermal adaptation induced substitution biases. A web server implementing the scoring function and the dataset used in this study are freely available at http://www.abl.ku.edu/thermorank/.

## Background

Developing thermostable proteins has been a main focus of protein engineering because of its theoretical and practical significance [1-4]. Recently, computational protein design methods have been attracted much attention due to their potential cost and time savings over conventional directed evolution approaches [3,5,6]. These types of approaches utilize information extracted from protein sequences and/or 3D structures to predict favorable mutations that may enhance protein thermostability. Clearly, a key step in such approaches is the development of reliable methods for estimating the relative stability of possible mutants to identify favorable mutations. Such methods may also help better understand the protein-folding problem since the ultimate outcome of protein folding is a native structure with the lowest free energy among many possible structures of a protein.

A common approach to study the thermostability of proteins is to perform comparative studies of the sequences and/or structures of (hyper)thermophilic proteins (HPs) and their mesophilic counterparts (MPs) [7-15] because there exists a direct positive correlation between the optimal growth temperature (OGT) of an organism and the melting temperature of its proteins, a key metric of protein thermostability [16,17]. Numerous studies have focused on amino acid composition changes caused by thermal adaptation at the whole genome level [7,14,18]. For example, Zeldovich *et al*. discovered that the total concentration of seven amino acids (INYWREL) in the proteins of an organism has a strong correlation with its OGT [14]. Overall, the proteins of thermophiles contain more charged and hydrophobic amino acid residues at the expense of polar ones [7,14,18]. The observed composition differences have prompted the development of predictive models discriminating HPs and MPs [19-21]. For example, Gromiha and Suresh applied 12 different classification algorithms and the best accuracy achieved reached 89% [21].

Several amino acid substitution preference matrices have been created based on the sequence alignments of thermophilic proteins and their mesophilic homologues [22-24]. Analyzing these matrices and comparing sequences and structures of HPs and MPs have revealed a number of substitution trends potentially affecting thermostability [7,8]. Notable features include: an increased level of charged residues in hyperthermophilic proteins at the cost of polar residues on surface compared to their mesophilic homologs [23,25,26]; elevated levels of proline or β-branched amino acids in loops to reduce the freedom of coil regions [1,27]; a reduced number of residues in coil regions but increases in helix runs [28,29]; increased numbers of the high helix-propensity residues such as Lys and Glu, etc. [30]; an increased compactness of hydrophobic cores resulted in enhanced apolar interactions and interior packing [30-32]; and reduced deamidation probability by replacing Gln with Glu and Asn with Asp [33,34].

The goal of this study was to develop a scoring function for predicting relative thermostability of protein and their mutants using an integrated statistical and machine learning approach. We used HP/MP orthologs as research subjects because they are equivalent to mutants with multiple substitutions and, as discussed above, the difference between them may encode thermal-adaptation mechanisms. Thus a scoring function which can distinguish HP/MP orthologs is presumably able to rank the relative stability of a protein and its mutants, a key step for designing more thermostable proteins.

In this study, we first constructed a set of 540 non-redundant hyperthermophilic-mesophilic protein ortholog pairs. Since our dataset is significantly bigger than previous studies, we then calculated a substitution preference matrix using an established approach [11,12,22-24]. We used a feature selection procedure based on the random forest algorithm to identify sequence-based features important to pairwise discrimination of hyperthermophilic and mesophilic protein orthologs. We then used a hill-climbing algorithm to fit a scoring function based on a linear combination of these important discriminating features. Finally, we applied the scoring function to two experimental datasets to demonstrate that this scoring function can indeed be used to rank thermostability of protein mutants with high accuracy.

## Methods

### Datasets

We downloaded all protein sequences of nine organisms, including four hyperthermophilic and five mesophilic organisms (Table 1) from the NCBI http://www.ncbi.nlm.nih.gov/. To identify HP-MP ortholog pairs, we performed BLAST searches for all MP sequences against all HP sequences [35]. The following conservative criteria were used to identify putative orthologs:

**Table 1:** The list of organisms whose proteins were used to generate the non-redundant hyperthermophilic (upper) and mesophilic (bottom) orthologous pairs (adopted from [48]).

Organism	Number of proteins	OGT (°C)
Aquifex aeolicus VF5	1560	96
Methanocaldococcus jannaschii DSM	1786	85
Thermotoga maritima MSB8	1858	80
Pyrococcus abyssi GE5	1898	103

Corynebacterium glutamicus ATCC	2993	30 - 40
Escherichia coli K12	4237	37
Mycobacterium tuberculosis H37Rv	3991	37
Bacillus halodurans C-125	4066	25 - 35
Streptococcus pneumoniae TIGR4	2094	30 - 35

• Reciprocal best BLAST hits with the e-values in BLAST searches less than 10^-10^;

• The difference in the number of residues is less than 5% of the shorter sequence so that only small insertions/deletions were allowed;

• Higher than 30% amino acid sequence identity.

In addition, we removed transmembrane proteins, predicted by TMHMM 2.0 http://www.cbs.dtu.dk/services/TMHMM/, because they often use different strategies from soluble proteins to survive under high temperature environments [36]. Furthermore, to reduce the statistical bias caused by redundancy, we clustered paralogues using the blastclust program available in the BLAST package [35]. The minimum length coverage of blastclust was set to 0.5 and the sequence similarity threshold was set to 0.25. Sequences longer than 600 or shorter than 50 residues were also removed. The final dataset consists of 540 non-redundant HP-MP ortholog pairs. Pfam http://pfam.sanger.ac.uk/ domain scans of these proteins confirmed, as expected, that the two proteins of each ortholog pair contain the same domains. Thus the selected pairs are very likely true orthologs.

We also used a set of 373 structurally well-aligned protein pairs from (hyper)thermophilic and mesophilic organisms compiled by *Glyakina et al*. for testing purpose [37]. The dataset includes 63 hyperthermophilic and 310 thermophilic proteins.

### Amino acid substitution matrix

The amino acid residue substitution matrix was constructed following an established procedure [11,12,22-24]. In brief, we counted each of the 380 types of amino acid residue substitutions in the BLAST sequence alignments of all MP/HP pairs. Substitutions in converting MPs to HPs are considered as the "forward" direction. Two-tail binomial statistics were used to estimate the statistical significance of the asymmetry of the forward and reverse substitutions of any given pair of amino acids [23].

### Two sets of experimentally investigated protein mutations

In addition to the protein pairs mentioned before, we used two independent datasets for additional testing. The first set contains two wild-type adenylate kinases (ADKs) from *Methanococcus Voltae *and *Methanococcus Jannaschii*, and a series of chimeric proteins generated from these two enzymes [38]. These proteins share significant sequence identity but differ in their thermostability (Table 2). The second dataset was collected by Montanucci and colleagues [20]. It contains 10 wild type proteins and 14 mutants manifesting thermal stability changes (Table 3). All protein sequences in these two tests were subjected to BLAST searches against all sequences in the 540 ortholog pairs. Only one protein, BsCSP (GI: 16077975), showed greater than 25% similarity to one of the protein sequences in the 540 pairs. Thus, these proteins can be used as independent testing datasets.

**Table 2:** Two wild-type ADKs and a series of chimeric enzymes generated from these two enzymes[38].

Seq_ID	Comm_meso	Comm_hyp	T_m _(°C)	Ranking
MJA	0	62	103	8
V36J	9	53	98	7
J160V	9	53	96	6
JVJ	37	25	89	4
VJV	20	42	82.5	5
V160J	51	11	74	2
J36V	53	9	73	3
MVO	62	0	69	1

Comm_meso and comm_hyp are the counts of the identical residues in the MP sequence MVO and the HP sequence MJA, respectively. The last column is the relative stability ranked by our scoring function (from least to most stable).

**Table 3:** The ranking of relative thermostability of wild type proteins and their mutated sequences using the scoring function.

Protein name	length	T_m_(°C)	Ranking
Dmeh (GI: 640374)	51	49	1
Dmeh_UMC	51	99	2
Dmeh_UVF	51	99	3

BsCSP (GI: 16077975)	67	53.8	1
BsCSP_mt1	67	69.7	2
BsCSP_mt2	67	83.7	3

PhyA (GI: 464382)	467	55	1
PhyA_mt18	467	62	2
PhyA_mt24	467	62+	3

PTDH (GI: 194552172)	336	39	1
PTDH_12x	336	59.7	2
PTDH_opt14	336	64.4	3

CbADH (GI: 187935035)	351	64.5	1
cbADH_Q100P	351	76	2

β-GUS (GI: 868020)	602	45	1
β-GUS_TR3337	602	65	2

FAOX (GI: 20302586)	372	37	1
FAOX_TE	372	45	2

Shble (GI: 3891709)	121	67.4	1
Shble_HTS	121	85.1	2

EcHPH (GI: 12539)	341	51	1
EcHPH_hph5	341	67	2

PDAO (GI: 129305)	347	45	2
PDAO_F42C	347	55	1

The data were originally collected by Montanucci *et al*. [20]. The sequence of cbADH was retrieved from the original literature by Goihberg *et al*.[49].

### Features

A set of 83 features derived from protein sequences was calculated using various software programs or in-house scripts (Table 4, more information about these feature is available in the additional file 1). These features can be roughly classified into two groups. The features in the first group, denoted as *c*_*k*_, are the absolute counts of amino acid residues or other properties. The features in the second group, labeled as *x*_*k*_, are the chain length normalized values of the features in the first group. Although including structure-based features may be of great help in understanding the mechanisms of mutagenesis induced protein stabilization, the vast majority of proteins lack solved structures. Therefore we only investigated the contributions from sequence-based features in this work. Furthermore, the theory that the sequence of a protein determines its structure suggests that the knowledge extracted from the sequence may be sufficient to distinguish proteins with different thermostability. Besides the general information extracted from the sequence, we also included several predicted features which were obtained by mature and widely-used algorithms, such as those used to predict secondary structure [39] and exposed/buried residues [40] (Table 4).

**Table 4:** The list of the 83 features used in the study.

Protein feature	Number of Features	Source
Sequence length (L)	1	In-house script

Count and composition of amino acids	40	In-house script

Number and percentage of positive, negative and all charged residues, as well as the net charges	8	In-house script

Number and percentage of small (T and D), tiny (G, A, S and P), aromatic (F, H, Y, W), aliphatic, hydrophobic and polar residues	12	In-house script

Number and percentage of residues which can form hydrogen bond in sidechain	2	In-house script

Number of sulfide atoms	1	In-house script

Average solubility of amino acids in aqueous solutions under room temperature	1	**

The average of the maximum solvent accessible surface area (ASA) of each amino acid	1	Eisenhaber[50]

Predicted isoelectric point (pI) of the protein, the average pI on all residues (pIa)	2	ProtParam[51]
	
Instability index and instability class	2	
	
Aliphatic index	1	
	
Gravy hydropathy index	1	

Composition of the predicted secondary structure residues	3	Psipred[52]

Predicted percentages of buried/exposed residues	2	Accpro[40]

The overall length and percentage of all coils, rem465, and hotloop	6	disEMBL[53]

**Obtained from The Merck Index, Merck & Co., Inc., Whitehouse Station, NJ 12 (1996).

### Random Forest

The random forest algorithm is an ensemble technique that utilizes the results of hundreds or even thousands of decision trees to perform classification or regression [41]. Each of the member trees is built on a bootstrap sample from the training data and utilizes a random subset of available variables. The algorithm has been applied in broad classification tasks and has frequently demonstrated superior performance compared to other classification algorithms [42,43]. It is robust and particularly suitable for classifying high-dimensional and noisy data. One very useful feature of the algorithm is that it offers several methods to assess the importance of various features based on their contributions to the correctness of the resulting classification [41]. In this study, we used the Gini importance to rank the importance of all used features. The Gini importance is the summation of the Gini impurity decreases in node splits made on the feature over all trees in the model. The Gini impurity is a common metric to measure the degree of impurity [44]. It is defined as:(1)

where *k *= (1, 2, ...,m) are possible classes and *p*_*k *_is the relative frequency of class *k *in a node A. Therefore *I*(A) equals to zero when all cases in the node belong to a single class and reaches its maximum when cases are equally distributed to all classes.

We used a random forest package implemented in the R environment for this study http://cran.r-project.org/web/packages/randomForest/index.html. Random forest models are usually insensitive to the model parameters [41]. Consequently the default parameters were used in the study.

## Results and Discussion

In this section, we first report a MP/HP residue substitution preference matrix generated from the BLAST pairwise alignments of MP and HP orthologs. Feature selection using the random forest algorithm is then described, followed by the scoring function construction. The performance of the scoring function in discriminating hyperthermophilic and mesophilic proteins was estimated with a set of holdout testing dataset. Finally, the application of the scoring function in predicting relative stability of proteins and their mutants is presented.

### Amino acid composition

The overall differences in amino acid composition between HPs and MPs are consistent with previous reports (Table 5) [7,11,22-24,45]. Based on the p-values from unpaired and paired t-test, the most significantly increased residues in HPs include Lys, Glu, Tyr, and Ile, while reduced residues include Gln, His, Ala, and Thr.

**Table 5:** Comparison of the composition of the amino acids in hyperthermophilic and mesophilic proteins and their significance p-values of *t*-test and paired *t*-test.

Amino acid	Composition in HP	Composition in MP	p-value (*t*-test)	p-value (paired *t*-test)
**S**	0.044 ± 0.016	0.050 ± 0.015	9.60×10^-9^	5.61×10^-12^

**Q**	0.019 ± 0.011	0.037 ± 0.015	6.81×10^-85^	1.24×10^-94^

**N**	0.035 ± 0.014	0.035 ± 0.015	0.88	0.85

**T**	0.042 ± 0.014	0.055 ± 0.016	1.02×10^-40^	2.44×10^-56^

**C**	0.009 ± 0.011	0.010 ± 0.011	0.36	0.08

**G**	0.075 ± 0.019	0.079 ± 0.020	9.14×10^-4^	9.68×10^-10^

**A**	0.066 ± 0.023	0.080 ± 0.028	3.41×10^-48^	1.08×10^-87^

**H**	0.017 ± 0.010	0.024 ± 0.013	3.00×10^-20^	4.64×10^-40^

**M**	0.024 ± 0.011	0.026 ± 0.010	0.02	3.00×10^-3^

**Y**	0.033 ± 0.014	0.027 ± 0.013	6.10×10^-15^	4.47×10^-31^

**F**	0.038 ± 0.015	0.033 ± 0.014	3.00×10^-8^	1.29×10^-14^

**V**	0.086 ± 0.021	0.082 ± 0.020	2.32×10^-4^	5.36×10^-7^

**L**	0.089 ± 0.021	0.089 ± 0.022	0.73	0.59

**P**	0.041 ± 0.015	0.040 ± 0.014	0.39	0.16

**I**	0.077 ± 0.020	0.066 ± 0.019	6.46×10^-20^	3.15×10^-29^

**W**	0.008 ± 0.007	0.007 ± 0.007	0.05	3.00×10^-3^

**D**	0.050 ± 0.015	0.057 ± 0.016	1.92×10^-12^	5.64×10^-22^

**E**	0.097 ± 0.023	0.079 ± 0.022	6.73×10^-38^	5.63×10^-75^

**K**	0.091 ± 0.023	0.060 ± 0.023	1.21×10^-87^	5.25×10^-117^

**R**	0.056 ± 0.023	0.055 ± 0.023	0.57	0.36

**Figure 1:**
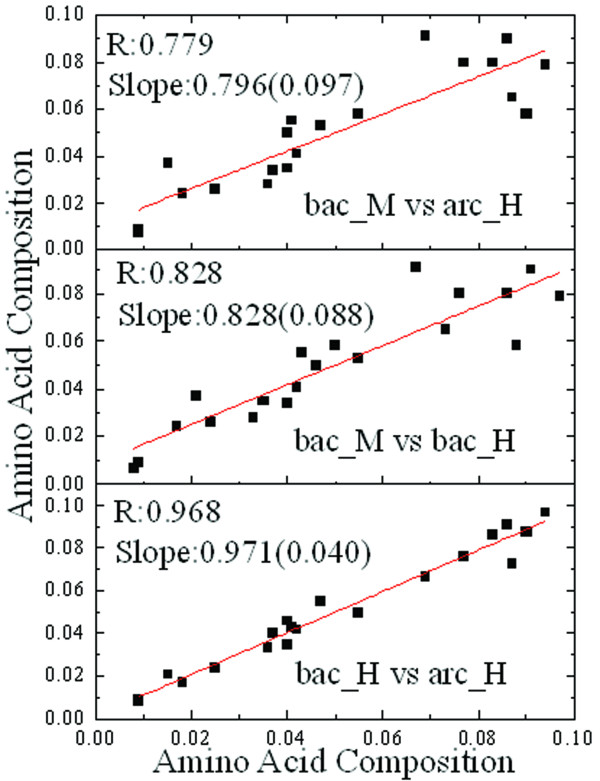
**The pariwise comparisons of amino acid compositions in the three different sets of proteins**. The solid lines show the best-fit of linear regression lines with regression coefficient and slope displayed and the dash lines show the orthogonal line. Bac_M, arc_H and bac_H are proteins from mesophilic bacteria, hyperthermophilic archaea and bacteria, respectively.

The 540 HPs consist of 426 bacteria and 114 archaea proteins while all MPs are from bacteria. In order to rule out the possibility that the different domains cause bias toward residue composition and the final results, we calculated the correlation coefficients of the amino acid compositions between HPs from archaea and MPs, HPs from bacteria and MPs, and HPs from bacteria and HPs from archaea. The R values of the correlations are 0.779, 0.828, and 0.968, respectively (Figure 1). Therefore, the composition difference possibly attributed to bacteria *vs*. archaea domains isn't as significant as the contributions by thermal adaptation.

### Amino acid residue substitutions

**Figure 2:**
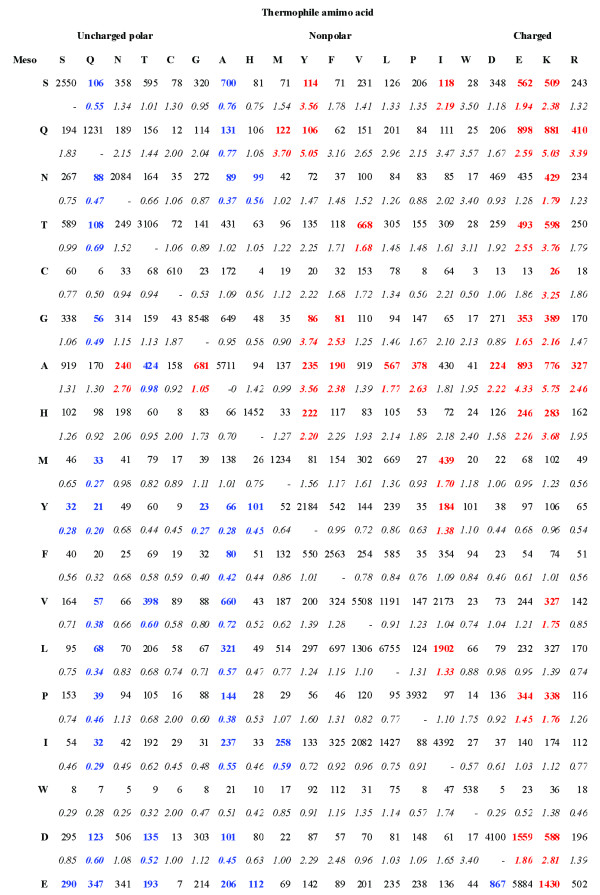
**Amino acid substitutions between mesophilic and hyperthermophilic proteins**. The top number in each cell is the observed substitution instances and the bottom one (in *italics*) is the ratio of the number of the substitution cases to the opposite substitution. Significant biased substitutions (p-value < 10^-10^, two-sided Fisher's exact test) are highlighted in bold. Red cells are significant HP favored substitutions while blues are MP favored.

All 380 residue substitutions are reported in Figure 2. We also calculated the ratio of each substitution to the opposite replacement. Substitutions with statistically significant bias (p < 10^-10^) are shown in bold. Red cells are substitutions favored in the MP to HP direction while blues are favored in the opposite direction. There are 84 (22%) significantly biased substitutions and 44 of them are in the direction from MP to HP. The overall trends of the substitution preferences are consistent with previous studies [22-24]. For example, charged residues, especially Lys and Glu, gain significantly in HPs at the cost of uncharged polar residues such as Ser, Gln, and Tyr.

Many of the significant substitution asymmetries are consistent with various proposed protein thermo stability mechanisms. For example, Asp is preferred to be substituted by Glu or Lys in the direction from MP to HP, both are helix favored while Asp is coil favored. This is consistent with previous findings that in general HPs contain more helical regions at the cost of disordered regions than MPs [28,29]. There is a strong preference for Ser, Thr, Asn and Gln to be substituted by Lys and Glu in HPs, which can be explained by the observed significant reduction of polar non-charged residues [23,26] and deamidation vulnerable residues [33,34] in HPs. Leu is preferred to be substituted by Ile to enhance thermo stability. This is consistent with the finding that increasing β-branched amino acids in loop regions enhance protein thermostability [1,27].

It is worth mentioning that the significance threshold (p < 10^-10^, Fisher's exact test [46]) used in this study was significantly more stringent than the criteria used in previous studies (e.g. p < 10^-2^) because we used approximately five times as many HP/MP pairs as previous studies. The ratios of forward-to-reverse changes for these substitutions were also calculated based on more examples than in previous studies. For example, the matrix reported by Haney *et al*. contained 72 residue replacements with no or only single instances [23]. In our matrix, the minimum number is 3 and there are only 14 substitutions with less than 10 examples. Therefore the ratios in this matrix may better reflect thermal adaptation induced substitution biases and should be useful in designing thermostable proteins.

### Ranking features using a random forest algorithm

The analysis of the residue substitution preference between MPs and HPs clearly indicates that different residues contribute to protein thermostability differentially. In this section, we describe a procedure for ranking the importance of all 83 features derived from protein sequences in discriminating MPs and HPs using the random forest algorithm.

**Figure 3:**
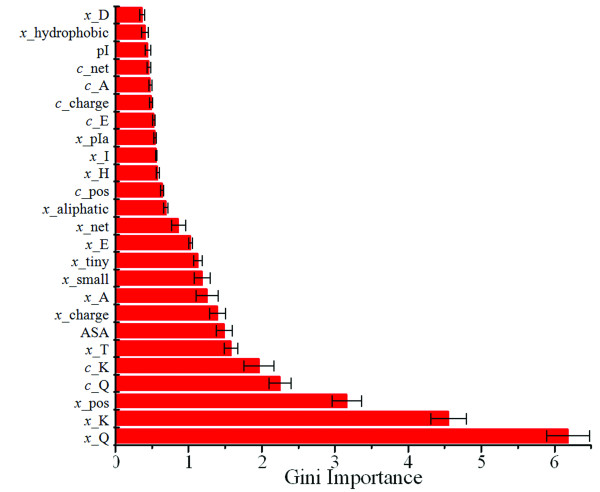
**The 25 most important features ranked by the Gini importance of the random forest algorithm**. The prefixes c_ and x_ of each feature indicate that the feature is an absolute count or normalized value, respectively.

A standard five-fold cross validation procedure was used to determine the importance of features and develop the scoring function. We randomly split the 540 pairs into five equal portions. We used four portions as training datasets and reserved the remaining portion for testing purposes. We then constructed a random forest model with 3000 trees for discriminating these 432 ortholog protein pairs in the training set and then used the Gini importance to rank these features. The procedure was repeated four more times and each time a different portion was used as the testing dataset. We found that the results from all five runs were very consistent. All features were ranked by their average importance and top 25 are shown in Figure 3. The levels of glutamine and lysine are most important among the 83 features used in this study, followed by the percentage of positively charged residues. We also noticed that the features normalized by sequence length are consistently more important than the corresponding absolute counts. Thus, we only used the normalized features in the scoring function. Interestingly, all predicted features, such as secondary structure, the ratio of exposed to buried residues, and the disordered region predictions, failed to appear in the 25 most important features.

### Developing the scoring function

**Figure 4:**
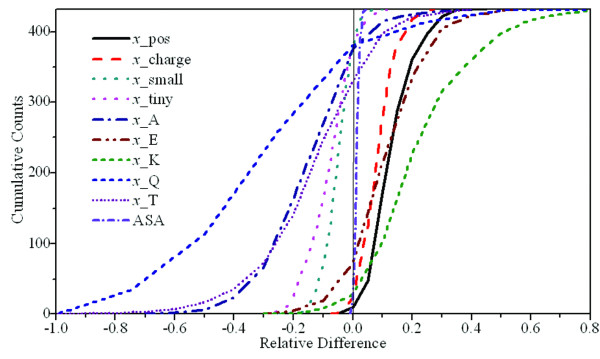
**The cumulative curves of the 10 most important features against the relative difference between hyperthermophilic and mesophilic sequences**.

We first calculated the relative feature difference Δ*x*_i_, which is defined as:(2)

where *x*_*i*_(seq1) and *x*_*i*_(seq2) are the values of the *i*th feature from the first sequence and the second sequence, respectively. We plotted the cumulative curves of the relative feature difference Δ*x*_i _of the ten most significant normalized features in the training dataset (Figure 4). In this plot, all cumulative curves show typical sigmoid shapes in which the inflexion points are located in the curve at the half height, i.e., the cumulative counts are equal to half of the total counts.

We constructed the scoring function by a linear combination of the ten most important features. The scoring function can be written as:(3)

where *i *runs over all 10 features are used in the scoring function and *w*_*i *_is the weight for each feature. The sign of the weight of each feature was determined by the location of the inflexion point of its cumulative curve: positive for features located to the left and negative for those to the right of the zero-difference line. Thus the signs of *x*_K, *x*_E, *x*_pos, *x*_charge, and ASA are positive, and negative for *x*_small, *x*_tiny, *x*_A, *x*_Q and *x*_T. We then used a hill-climbing algorithm to fit the weights of these features. The absolute values of all weights were restricted to the range of 0 to 1. We randomly assigned an initial weight to each feature and counted the number of correctly ranked ortholog pairs. The weights were then randomly updated and the number of correct ranks was recounted. The new weights were kept if they resulted in more correctly ranked ortholog pairs; otherwise the weights were rolled back to the previous values. This procedure was repeated 5 × 10^7 ^times and the batch of weights which maximized the number of positive score values was recorded. To check whether the optimization procedure was trapped in a local maximum, we repeated the procedure four more times using different random seeds. The results were very similar and thus we simply used the average of the weights in the scoring function. We then used the same procedure to develop four more scoring functions, each for one of remaining training datasets.

### The discrimination ability of the scoring function

We calculated the accuracies of the discriminations made by the five scoring functions on their corresponding training datasets. The scoring functions using optimized weights were able to distinguish in average 427.1 ± 1.9 out of 432 (98.9% accuracy) ortholog protein pairs in the training datasets. We then tested each of the scoring functions with its corresponding holdout testing dataset. Out of 108 protein pairs in the testing sets, on average 105.1 ± 0.5 pairs were correctly ranked (97.3% accuracy). This was very close to the accuracy obtained from the training sets (98.9%). Thus the scoring function is robust and able to discriminate a broad spectrum of HP and MP homologous protein pairs.

The average weights for each feature determined in all five training procedures are quite consistent. Thus we simply use the averages of these weights in the final scoring function (Table 6). It is noteworthy that while the signs of the weights indicate whether the features are favorable or not in hyperthermophilic proteins, their absolute values are not significant since the features are not normalized to a common scale.

**Table 6:** The final weights of the ten features used in the scoring function.

Feature	*x*_K	*x*_E	*x*_pos	*x*_charge	ASA	*x*_small	*x*_tiny	*x*_A	*x*_Q	*x*_T
**Weight**	0.75	0.20	0.80	0.20	0.90	-0.20	-0.20	-0.30	-0.10	-0.20

We also applied the scoring function to discriminating (hyper)thermophilic and mesophilic proteins in the Glyakina dataset [37]. Our scoring function was able to correctly discriminate not only 59 HP/MP pairs (93.7% accuracy), but also 238 thermophilic and mesophilic pairs (76.8% accuracy). The list of these proteins and their scores are provided in Table S2 in the additional file 1. We believe that the difference of the accuracy between hyperthermophilic and thermophilic proteins was caused by the different stabilization mechanisms of hyperthermophilic and thermophilic proteins, as previously suggested in literature [17,31].

### Discriminating non-homologous protein pairs

Encouraged by the results in the above test, we further challenged the scoring function in discriminating non-homologous HP/MP protein pairs. In this test, we compared each HP protein sequence against all MP sequences. The overall accuracy of these 540*540 pairwise comparisons was 88.4%. Such a high accuracy in discriminating non-homologous HP and MP sequences confirms that HP sequences share some common sequential patterns to generate sufficient stability at elevated temperature.

### Application in ranking the thermostability of proteins and their mutants

The first test was carried out on two wild-type ADKs and a series of chimeric enzymes generated from these two enzymes [38]. The predicted ranking of thermo-stability using the scoring function is highly consistent with the experimental results (Table 2). In all 28 () pairwise comparisons, only two resulted in incorrect predictions (92.9% accuracy). Moreover, the two inaccurate predictions included one between VJV and JVJ in which the Tm differed by only 6.5°C, and the other between V160J and J36V in which the Tm differed by just 1°C, probably not an experimentally detectable difference.

In the second test, we used a batch of sequences collected by Montanucci, *et al *[20]. The sequence lengths, the GI numbers of the wild-type proteins, and their melting temperatures are listed in Table 3. We used the scoring function to rank the relative thermostability of wild-type proteins and their mutants. In the case of proteins with two mutants, the relative stability of these mutants was also predicted. Overall there were 18 pairwise comparisons between these wild proteins and their mutants. The scoring function achieved an accuracy of 94.4% (17/18). The wrong prediction was for protein PDAO and its mutant (Table 3). It is a single mutation (F42C) and the difference in Tm is moderate (10°C).

Overall, the scoring function has consistently demonstrated a remarkable ability to rank the relative thermostability of proteins and their mutants. Thus a website http://www.abl.ku.edu/thermorank/ was created and made freely available to the general public.

### Comparison with other Methods

The current study differs at the level of information granules from previous work focused amino acid composition differences between thermophilic and mesophilic organisms [7,14,18]. We focused on the differences between HP and MP ortholog pairs instead of on the differences between thermophilic or mesophilic proteins at the genome level. The difference between these two approaches is similar to the one between unpaired and paired two-sample *t*-tests. While previous studies have succeeded in revealing the overall changes caused by thermal adaptation at the genome level, our study has further focused on the protein level. Such an approach may reduce or eliminate the effects of confounding factors such as protein families because it is well established that the amino acid composition may vary in different protein classes [47]. In addition, a protein level study may be more relevant to designing stable proteins because orthologs are essentially mutants with multiple mutations.

To compare the performance of our algorithm to other approaches is difficult because very few algorithms have been developed to rank the relative thermostability of HP/MP orthologous pairs and these studies have used different datasets [20,48]. TargetStar, a scoring function based on the analysis of 1006 decoy structures for a given protein, can discriminate HP/MP orthologs pairs with 77% accuracy [48]. Recently, Montanucci and colleagues reported a SVM model which achieves 88% accuracy on a set of redundancy-reduced HP/MP pairs [20]. The SVM model used residue and dipeptide compositions as predictive features. Thus, the 97.3% predictive accuracy on the test dataset of our scoring function is considerably higher than the reported accuracies of both previous methods. Moreover, in the application of predicting the relative thermostability of proteins and their mutants, our approach achieved an accuracy of 94.4% (17/18) in the second blind test set, which represents one more correct prediction than Montanucci *et al*. on the same dataset [20].

## Conclusions

We have presented a novel scoring function which can distinguish not only HP/MP ortholog pairs, but also non-homologous pairs at high accuracies. Most importantly, it can be used to accurately predict the relative stability of proteins and their mutants, as demonstrated in two blind tests. In addition, the residue substitution preference matrix assembled in this study may better reflect the thermal adaptation induced substitution biases than previous studies because a larger dataset was used. The large set of HP/MP is available in the supplementary website and should be useful to other researchers for further development of novel algorithms in this area.

## Abbreviations

HP: hyperthermophilic protein; MP: mesophilic protein; OGT: optimal growth temperature; Tm: melting temperature.

## Authors' contributions

JWF conceived the project. YL and JWF carried out the study with input from CRM. YL, CRM and JWF drafted the manuscript. All authors have read and approved the final manuscript.

## Supplementary Material

Additional file 1 **Supplementary Table S1, S2, and Figure S1**. This file contains the following contents: 1. Table S1. Detailed description of the 10 features used to construct the scoring function. 2. Table S2. A test of the discriminative ability for the scoring function on a dataset containing 63 hyperthermophilic-mesophilic protein pairs and 310 thermophilic-mesophilic protein pairs. 3. Figure S1. The ROC curve of the scoring function in discrimination of 540 pairs of ortholog protein sequences accumulated from the 5-fold cross testing set. Click here for file
