# Supplementary material for: A novel scoring function for discriminating hyperthermophilic and mesophilic proteins with application to predicting relative thermostability of protein mutants
Source: BMC Bioinformatics. 2010 Jan 28;11:62. doi: 10.1186/1471-2105-11-62 (PMC3098108; doi:10.1186/1471-2105-11-62)

Additional File 1

Contents

1. Table S1. Detailed description of the 10 features used to construct the scoring function.
2. Table S2. A test of the discriminative ability for the scoring function on a dataset containing 63 hyperthermophilic-mesophilic protein pairs and 310 thermophilic-mesophilic protein pairs.
3. Figure S1. The ROC curve of the scoring function in discrimination of 540 pairs of ortholog protein sequences accumulated from the 5-fold cross testing set.

Table S1. Detailed description of the 10 features used in the scoring function.

Protein features	Feature description
*x*_pos	The composition of positively charged residues R, K and H
*x*_charge	The composition of charged residues R, K, H, D and E
*x*_small	The composition of small residues T and D
*x*_tiny	The composition of tiny residues A, G, P and S
*x*_A	The composition of residue A
*x*_E	The composition of residue E
*x*_K	The composition of residue K
*x*_Q	The composition of residue Q
*x*_T	The composition of residue T
ASA	The average of the maximum solvent accessible surface area of each amino acid*

* Frank Eisenhaber PA: **Improved strategy in analytic surface calculation for molecular systems: Handling of singularities and computational efficiency**. *Journal of Computational Chemistry* 1993, **14**(11):1272-1280.

Table S2. A test of the discriminative ability for the scoring function on the dataset containing 63 hyperthermophilic-mesophilic protein pairs and 310 thermophilic-mesophilic protein pairs from the 373 well culled homologous pairs by Glyakina AV, et al. Different packing of external residues can explain differences in the thermostability of proteins from thermophilic and mesophilic organisms. Bioinformatics 2007, 23(17): 2231-2238.

Part A. 63 hyperthermophilic-mesophilic protein pairs, 59 pairs were correctly predicted.

Hyperthermophilic	Mesophilic	Score_value	Correct?
1wdvA	1dbuA	-0.353	NO
2cv4A	1prxA	-0.463	NO
1mowA	1g9zA	0.060	YES
1hv8A	1xtiA	0.439	YES
1ki9A	1khtA	0.344	YES
1rqgA	1qqtA	0.874	YES
1rqgA	1qqtA	0.874	YES
1pznA	1xu4A	0.246	YES
1aj8A	1a59A	1.091	YES
1xi3A	2tpsA	0.481	YES
1bq8A	1iroA	0.259	YES
1sj1A	1fxdA	0.599	YES
1e19A	1b7bA	0.752	YES
1xi8A	1g8lA	1.663	YES
1xx7A	2paqB	0.608	YES
1s4eF	1pieA	0.654	YES
1s4eF	1pieA	0.654	YES
1a1sA	1dxhA	-0.034	NO
1a8lA	1hyuA	0.670	YES
1v8zA	1a5aB	0.686	YES
1xgsA	1r58A	0.415	YES
1jg2A	1i1nA	0.938	YES
1geqB	1rd5A	0.812	YES
1iqpA	1sxjB	0.360	YES
1xexB	1w1wB	0.289	YES
1v3wA	1xhdA	0.340	YES
1uddA	1yafA	0.575	YES
1v8aA	1c3qA	0.929	YES
1im5A	1j2rA	1.502	YES
1v30A	1xhsA	2.313	YES
1v6tA	1xw8A	1.512	YES
1wlsA	1nnsA	1.111	YES
1wlsA	1nnsA	1.111	YES
2bj7A	1q5yA	1.118	YES
1lk5A	1m0sA	0.344	YES
1gd9A	1j32A	1.043	YES
1iq8A	1wkeA	1.113	YES
1w2iA	2acyA	0.074	YES
1j0aA	1f2dA	0.499	YES
1iz6A	1xtdA	0.742	YES
1d3uB	1c9bA	0.824	YES
1d3uB	1c9bA	0.824	YES

1qypA	1tfiA	-0.208	NO
1a2zA	1augA	0.460	YES
1ny5A	1peyA	0.778	YES
1hqkA	1rvv1	0.505	YES
1fxqA	1g7vA	0.374	YES
1pybA	3ersX	0.703	YES
1oz9A	1xm5A	2.238	YES
1l8qA	1j1vA	0.787	YES
1oy5A	1p9pA	1.575	YES
1ulzA	1dv1A	0.430	YES
1ulzA	1dv1A	0.430	YES
1ulzA	1dv1A	0.430	YES
1wy5A	1ni5A	2.567	YES
1lfpA	1mw7A	0.242	YES
1lfpA	1mw7A	0.242	YES
1udnA	1r6lA	0.765	YES
1udnA	1r6lA	0.765	YES
1ny5A	1ojlB	1.810	YES
1ny5A	1ojlB	1.810	YES
1c3rA	1t64A	0.968	YES
1wwrC	1p6oA	0.692	YES

Part B. Out of a total of 310 thermophilic-mesophilic protein pairs, 238 pairs were correctly predicted.

Thermophilic	Mesophilic	Score_value	Correct?
1m5hA	1m5sA	0.061	YES
1q6wA	1iq6A	0.679	YES
1hjzA	1spvA	1.780	YES
1jnrA	1nekA	0.692	YES
1omoA	1x7dA	0.658	YES
1jjiA	1lzlA	1.479	YES
1p1lA	1oscA	0.873	YES
1rxvA	1ul1Z	-0.202	NO
1rxvA	1ul1Z	-0.202	NO
1rwzA	1sxjG	0.472	YES
1rwzA	1sxjG	0.472	YES
1j2pA	1rypG	-0.029	NO
1sauA	1yx3A	0.824	YES
1y1lA	1ljlA	0.367	YES
1u1iA	1p1jA	0.407	YES
1vimD	1m3sB	0.586	YES
1gkuB	1mw8X	0.394	YES
1t7lA	1u1hA	0.824	YES
1t7lA	1u1hA	0.824	YES
1mroA	1e6yA	-0.175	NO
1mroC	1e6yC	0.434	YES
1hbnB	1e6yB	0.269	YES
1hbnB	1e6yB	0.269	YES
1gtdA	1t4aA	-1.098	NO
1ep0A	1upiA	1.798	YES
1g5cA	1ylkA	0.837	YES
1lvwA	1mp3A	0.470	YES
1lxnA	1lxjA	-0.368	NO
1km2A	1eixA	0.231	YES
1lssA	1lsuA	0.403	YES

1g9xA	1g6hA	-0.007	NO
1nvtA	1npdA	0.917	YES
1nvtA	1npdA	0.917	YES
1xhkA	1rr9D	0.420	YES
1twiA	1hkvA	1.543	YES
1fszA	1ofuA	0.977	YES
1fszA	1ofuA	0.977	YES
1f5sA	1l8lB	1.278	YES
1g0hA	1imbA	1.066	YES
1g0hA	1imbA	1.066	YES
1snnA	1k4iA	1.208	YES
1g61A	1g62A	1.712	YES
1go3M	1y14D	0.673	YES
1b78A	1k7kA	1.478	YES
1qztA	1td9A	-0.154	NO
1lojA	1n9rA	0.449	YES
1nj1A	1nyrA	-0.247	NO
1lnqA	1id1A	-0.500	NO
1mgtA	1sfeA	1.729	YES
1pg5B	1nbeB	0.634	YES
1pg5B	1nbeB	0.634	YES
1mp9A	1qnaB	0.076	YES
1qdlB	1i7qB	1.625	YES
1xttA	1bd3A	0.122	YES
1vphA	1xbfA	-0.137	NO
1v4nA	1cb0A	0.396	YES
1nogA	1rtyA	0.579	YES
1rlkA	1q7sA	0.611	YES
1urdA	3mbpA	-1.081	NO
1n1qA	1jigA	-0.440	NO
1amuA	1mdbA	0.306	YES
1amuA	1mdbA	0.306	YES
1amuA	1mdbA	0.306	YES
2cevA	1t4tA	-0.221	NO
1i5fA	1mjcA	0.393	YES
1r0rE	1sbhA	-0.189	NO
1gbgA	2ayhA	-0.148	NO
1i2sA	1ylpA	1.290	YES
1nrfA	1xa1B	-1.082	NO
1p6rA	1okrB	-0.100	NO
1skyB	1w0jA	-0.477	NO
1skyB	1w0jA	-0.477	NO
1skyB	1w0jA	-0.477	NO
3pvaA	2bjfA	-0.490	NO
1zinA	1s3gA	0.000	YES
1tilF	1auzA	-0.493	NO
1b4aA	1f9nE	-0.128	NO
1fc3A	1lq1C	0.103	YES
1gtfA	1wapA	-0.176	NO
1knvA	1cfrA	0.804	YES
1i6mA	1yi8B	0.860	YES
1b04A	1ta8A	-0.184	NO
1u4bA	2kfnA	0.120	YES
2ts1A	1x8xA	0.286	YES
2pjrA	1uaaA	0.115	YES
2pjrA	1uaaA	0.115	YES

2pjrA	1uaaA	0.115	YES
1g2wA	1iyeA	1.099	YES
1g2wA	1iyeA	1.099	YES
1rrsA	1wefA	0.350	YES
1seiA	1s03G	0.340	YES
1brwB	2tptA	0.459	YES
1brwB	2tptA	0.459	YES
1kkjA	1eqbB	-0.364	NO
1lqyA	1lm4B	-0.597	NO
4pfkA	1pfkA	0.144	YES
1y51A	1sphA	0.138	YES
1tigA	2ifeA	-0.286	NO
1wp5A	1zi0B	0.757	YES
1r2zA	1pjjA	-0.953	NO
1r2zA	1pjjA	-0.953	NO
1r2zA	1pjjA	-0.953	NO
1rfzA	1y9iA	-0.042	NO
1ebdA	1lvlA	0.381	YES
1ebdA	1lvlA	0.381	YES
1x87A	1uwlA	0.276	YES
1t8hA	1rw0A	-0.505	NO
1miwA	1ou5A	-0.477	NO
1u9cA	1rw7A	-0.265	NO
1phpA	1hdiA	0.381	YES
1phpA	1hdiA	0.381	YES
1keiA	1espA	-0.054	NO
1su7A	1jqkA	0.282	YES
1su7A	1jqkA	0.282	YES
1su7A	1jqkA	0.282	YES
1ov8A	1cuoA	-2.911	NO
2bm3A	1qznA	0.846	YES
1anuA	1g1kA	0.193	YES
1nbcA	1g43A	0.794	YES
1ybxA	1pugA	0.224	YES
1xquA	1kpfA	-0.085	NO
1xrgA	1oniA	0.904	YES
1f5jA	1igoA	-1.279	NO
1mqqA	1gqiA	0.465	YES
1ynrA	351cA	0.781	YES
1rfkB	1czpA	-0.152	NO
1vf5A	1q90B	0.307	YES
1vf5C	1q90A	-0.432	NO
1ycgA	1e5dA	0.068	YES
1ycgA	1e5dA	0.068	YES
1y80A	1bmtA	-0.535	NO
1bawA	7pcyA	0.993	YES
1ugsA	1ahjA	1.162	YES
1h0bA	2nlrA	1.331	YES
1rblA	1rboB	0.128	YES
1rblA	1rboB	0.128	YES
1ktpA	1jboA	0.001	YES
1obrA	1m4lA	0.098	YES
1yfzA	1tc1B	0.770	YES
1lf6A	1ulvA	1.626	YES
1ykfA	1jqbA	-0.017	NO
1iuaA	1ckuA	0.109	YES

1eysM	1dxrM	0.279	YES
1eysC	1prcC	-0.114	NO
1eysH	1prcH	0.529	YES
1tf4A	1g87B	-0.539	NO
1mz4A	1f1cA	0.518	YES
1v2zA	1r5qA	0.328	YES
1o4vA	1xmpC	0.451	YES
1inlC	1iy9A	0.055	YES
1wosA	1yx2B	0.222	YES
1wosA	1yx2B	0.222	YES
1nz0A	1a6fA	0.750	YES
1o12A	2vhlB	0.907	YES
1u0lA	1t9hA	0.559	YES
1u0lA	1t9hA	0.559	YES
1vlqA	1l7aA	0.202	YES
1vq0B	1vzyA	0.811	YES
1vq0B	1vzyA	0.811	YES
1ww1A	1y44B	0.583	YES
2a61A	1s3jA	1.087	YES
1vl4A	1vpbA	0.465	YES
1vmaA	1ftsA	0.464	YES
1vmaA	1ftsA	0.464	YES
1dd3A	1ctfA	-0.393	NO
1sg9A	1t43A	2.274	YES
1sg9A	1t43A	2.274	YES
1r3eA	1k8wA	1.244	YES
1wa3A	1euaA	1.280	YES
1eg5A	1p3wA	0.531	YES
1j6oA	1xwyA	1.599	YES
1l1jA	1te0A	1.026	YES
1m4yA	1e94A	-0.098	NO
1mkmA	1tf1A	2.144	YES
1o1xA	1nn4B	0.629	YES
1o6dA	1ns5A	1.262	YES
1pvtA	1gt7A	1.645	YES
1vkzA	1gsoA	1.223	YES
1vlaA	1ml8A	1.224	YES
1vmdB	1s89B	1.622	YES
1vpaA	1i52A	1.502	YES
1j6uA	1gqyB	0.731	YES
1j6uA	1gqyB	0.731	YES
1o5zA	1jbwA	1.740	YES
1hh2P	1k0rA	1.312	YES
1hh2P	1k0rA	1.312	YES
1o54A	1i9gA	1.785	YES
1o54A	1i9gA	1.785	YES
1o4uB	1qpoA	1.666	YES
1o4uB	1qpoA	1.666	YES
1tzvA	1eyvA	1.890	YES
2btyA	2bufA	0.859	YES
1tqgA	1i5nC	1.464	YES
1rq0B	1zbtA	0.370	YES
1vjrA	1ys9A	1.526	YES
1vknA	1xygA	0.550	YES
1cz3A	1qzfA	0.499	YES
1q7zA	1lt8A	0.480	YES

1o0wA	2a11A	1.766	YES
1o20A	1vluA	0.532	YES
1vl1A	1y89A	1.325	YES
1up7A	1u8xX	0.951	YES
1up7A	1u8xX	0.951	YES
1vpqA	1vpyA	0.829	YES
1vljA	1oj7A	1.058	YES
1vljA	1oj7A	1.058	YES
1vm7B	1rkdA	1.227	YES
1vlgA	1eumA	0.601	YES
1vkuA	1t8kA	2.253	YES
1vpkA	1ok7A	0.829	YES
1vpkA	1ok7A	0.829	YES
1vpkA	1ok7A	0.829	YES
1z85B	1nxzA	0.744	YES
1usyE	1nh8A	1.624	YES
1vrgA	1on3A	0.607	YES
1vrgA	1on3A	0.607	YES
1zh8A	1h6dB	1.018	YES
1z82A	1n1eA	0.455	YES
1z82A	1n1eA	0.455	YES
1vp5A	1vbjA	0.190	YES
1vlhC	1qjcA	1.414	YES
1h98A	7fd1A	0.451	YES
1ewqA	1w7aA	0.265	YES
1ewqA	1w7aA	0.265	YES
1ewqA	1w7aA	0.265	YES
1rvgB	1gvfB	0.477	YES
1bxbA	2gyiA	0.717	YES
1bdmB	1b8pA	0.339	YES
1bdmB	1b8pA	0.339	YES
1vbiA	1z2iA	0.766	YES
3mdsA	1gv3A	0.784	YES
1udxA	1lnzA	-0.327	NO
1odeA	1dbfA	-1.380	NO
1v4vA	1o6cB	-0.348	NO
1v8qA	1y69U	-0.472	NO
1wkiA	1y69K	-0.225	NO
1ipdA	1cm7A	0.459	YES
1aipA	1efuA	-0.173	NO
1aipA	1efuA	-0.173	NO
1we3A	1pcqA	0.446	YES
1vc4A	1piiA	0.523	YES
1uluB	1qsgA	0.188	YES
1l0wA	1il2A	0.315	YES
1l0wA	1il2A	0.315	YES
1iw7A	1bdfA	0.226	YES
2prdA	1i40A	-0.061	NO
1v5xA	1piiA	0.482	YES
1iv3A	1h48A	-0.141	NO
1iy2A	1lv7A	0.296	YES
1iy2A	1lv7A	0.296	YES
1odlA	1k9sA	-1.172	NO
1korB	1k92A	0.540	YES
1korB	1k92A	0.540	YES
1vcoA	1s1mB	0.163	YES

1vcoA	1s1mB	0.163	YES
1we3O	1pcqO	0.380	YES
1adjA	1kmmA	0.931	YES
1ixrA	1d8lA	-0.261	NO
1ixrA	1d8lA	-0.261	NO
1j1yA	1psuA	0.151	YES
1j3bA	1oenA	0.361	YES
1j3bA	1oenA	0.361	YES
1kijA	1ei1A	0.064	YES
1kijA	1ei1A	0.064	YES
1oi7A	1jkjA	-0.087	NO
1qvrA	1khyD	1.800	YES
1qvrA	1r6bX	0.385	YES
1qvrA	1r6bX	0.385	YES
1ub7A	1hnjA	0.373	YES
1uekA	1oj4A	-0.180	NO
1uf9A	1viyA	1.394	YES
1ukkA	1nyeA	0.329	YES
1v93A	1b5tA	0.099	YES
1wubA	1y0gA	-0.655	NO
1ve1A	1y7lA	0.153	YES
1v8mA	1mqwA	2.089	YES
1v8fA	1mopA	1.116	YES
1j3lD	1nxjA	1.411	YES
1wkcA	1sbqA	-0.474	NO
1iugA	1vjoA	0.611	YES
1v9cB	1f2vA	0.447	YES
1ve2B	1s4dB	0.602	YES
1umdA	1qs0A	0.828	YES
1j33A	1l5oA	0.202	YES
1iqrA	1tezB	0.866	YES
1j3nA	1e5mA	0.126	YES
1j3nA	1e5mA	0.126	YES
1eh1A	1is1A	-0.150	NO
1ulqA	1m3kA	0.168	YES
1wekA	2a33B	-0.720	NO
1uarA	1orbA	0.495	YES
1uarA	1orbA	0.495	YES
1yyaA	1tph1	-0.350	NO
1umdB	1x7yB	0.019	YES
1umdB	1x7yB	0.019	YES
1atiA	1g5hA	0.070	YES
1v47A	1i2dA	0.292	YES
1v47A	1i2dA	0.292	YES
1onlA	1dxmA	-0.633	NO
1uiyA	1dciA	-0.037	NO
1usoA	1dcoC	0.177	YES
1fnmA	1zm9A	0.006	YES
1ka9H	1ox6A	-0.453	NO
1iq0A	1f7uA	-0.710	NO
1ukwA	3mddA	0.136	YES
1ukwA	3mddA	0.136	YES
1ukwA	3mddA	0.136	YES
1wx0A	1l6wA	0.459	YES
1tuxA	1ta3B	-0.193	NO
1gt6A	4tglA	0.139	YES

Figure S1. The ROC curve of the scoring function in discriminating 540 pairs of ortholog protein sequences accumulated from the testing sets in the 5-fold cross validation.

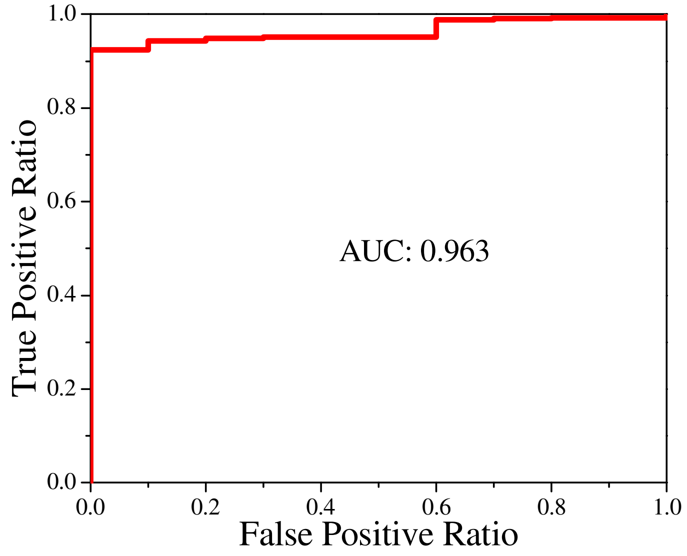

Supplement: Additional file 1 — Supplementary Table S1, S2, and Figure S1. This file contains the following contents: 1. Table S1. Detailed description of the 10 features used to construct the scoring function. 2. Table S2. A test of the discriminative ability for the scoring function on a dataset containing 63 hyperthermophilic-mesophilic protein pairs and 310 thermophilic-mesophilic protein pairs. 3. Figure S1. The ROC curve of the scoring function in discrimination of 540 pairs of ortholog protein sequences accumulated from the 5-fold cross testing set. [file 1471-2105-11-62-S1.PDF]
